# Non-targeted metabolomic analysis of follicular fluid in infertile individuals with poor ovarian response

**DOI:** 10.3389/fendo.2025.1547550

**Published:** 2025-05-12

**Authors:** Liang Guo, Jiaming Song, Xiyang Xia, Jianya Jiang, Yingying Yang, Wei Chen, Li Chen, Pingping Xue

**Affiliations:** ^1^ Department of Reproductive Medicine Center, Changzhou Maternal and Child Health Care Hospital, Changzhou Medical Center, Nanjing Medical University, Changzhou, China; ^2^ Changzhou Key Laboratory of Maternal and Child Health Medicine, Changzhou Maternal and Child Health Care Hospital, Changzhou Medical Center, Nanjing Medical University, Changzhou, China

**Keywords:** nontargeted metabolomics, poor ovarian response, follicular fluid, oocytes, metabolomics

## Abstract

**Background:**

Poor ovarian response (POR) is a pathological condition characterized by inadequate ovarian response to gonadotropin stimulation in patients undergoing *in vitro* fertilization and embryo transfer. It represents a primary cause of failure in many assisted reproductive technology treatments. Utilizing non-targeted metabolomics technology applied to follicular fluid, this research aims to elucidate the metabolic characteristics associated with POR, explore the underlying molecular mechanisms, and identify potential biomarkers. By analyzing metabolic factors that influence oocyte quality, we aspire to provide insights for the early detection and intervention of patients with POR.

**Methods:**

In this research, 60 follicular fluid samples were collected for a non-targeted metabolomic study, including 30 samples from POR patients and 30 from women with normal ovarian reserve. The orthogonal partial least squares discriminant analysis model was employed to discern separation trends between the two groups. Pathway enrichment analysis was performed using the Kyoto Encyclopedia of Genes and Genomes (KEGG) database. Additionally, random forest and logistic regression models were utilized to identify biomarkers indicative of POR within the follicular fluid.

**Results:**

Based on data from the Human Metabolome Database, our metabolomic analysis identified 40 differential metabolites associated with POR, including 18 up-regulated and 22 down-regulated metabolites. KEGG pathway analysis revealed that these metabolites predominantly participate in glycerophospholipid metabolism, choline metabolism in cancer, autophagy processes. Notably, perillyl aldehyde emerged as a potential biomarker for POR.

**Conclusions:**

This study represents the first comprehensive examination of metabolic alterations in follicular fluid among patients with POR using non-targeted metabolomics technology. We have identified significant metabolic changes within the follicular fluid of individuals affected by POR which may offer valuable insights into therapeutic strategies for managing this condition as well as improving outcomes in assisted reproductive technologies.

## 1 Introduction

Nowadays, infertility affects 20-30% of women of childbearing age (1). Despite the rapid advancements in assisted reproductive technology (ART) in recent years, a significant number of women exhibit poor responses to ovulation induction medications due to factors such as age, leading to unsatisfactory ART outcomes. Poor ovarian response (POR) is defined as a pathological condition wherein the ovaries inadequately respond to stimulation by gonadotrophins during *in vitro* fertilization and embryo transfer procedures. The main clinical manifestations include fewer developing follicles during the ovarian stimulation cycle, increased dosages of gonadotrophins required, and lower estrogen peaks (2). Statistics indicate that the incidence of POR ranges from 5.6% to 35.1% (3), accounting for approximately 9-26% of indications for *in vitro* fertilization (IVF) (4). It is the predominant cause behind many IVF failures (5). Due to its characteristics—such as high cycle cancellation rates, low oocyte retrieval numbers and diminished clinical pregnancy rates—POR presents significant challenges within ART development and has garnered considerable attention from experts in the field. To enhance diagnosis and treatment strategies for POR, various criteria have been proposed globally, including the Bologna criteria (6) and those established by the POSEIDON group (3). Nevertheless, controversy still surrounds the diagnostic approach for POR.

The metabolome comprises endogenous small molecules that participate in metabolism and maintain normal growth function of organisms. It is located in the downstream of gene regulatory and protein interaction network, and can provide terminal information. Metabolomics is dedicated to studying alterations within the metabolome across biological systems; this discipline offers direct and comprehensive insights into both physiological and pathological conditions present within an organism's body (7). Due to its capacity to amplify subtle changes in gene and protein expression at the functional level, with non-functional changes will not be reflected, metabolomics has garnered significant interest by scholars worldwide. The research utilizing metabolomics analysis technology in the field of reproduction is on the rise. Metabolomics is increasingly recognized as a vital research methodology within reproductive medicine. Currently, metabolomics analysis can be applied to investigate various biological fluids in women, including follicular fluid (FF) and embryo culture fluid. The composition of FF closely resembles that of plasma (8), reflecting the metabolic activities of granulosa cells, theca cells and other cells in the ovary. This unique microenvironment plays a crucial role in oocyte growth and significantly influences oocyte maturation (9). Consequently, metabolomic studies focusing on FF have emerged as a prominent topic within reproductive medicine. Numerous researchers have conducted metabolomics investigations on FF from patients with polycystic ovary syndrome, endometriosis and diminished ovarian reserve. These studies have yielded diverse conclusions regarding protein and lipid metabolites' expression levels, elucidating the pathophysiological process underlying these conditions from multiple perspectives (10, 11). The differential alterations observed in various metabolites within FF provide an intuitive reflection of follicular growth and development, thereby facilitating exploration into factors influencing oogenesis.

At present, there are relatively few studies examining the metabolomics of FF in patients with POR. Existing research typically follows clinical interventions; thus far, systematic non-targeted metabolomic analyses comparing POR individuals to those with normal ovarian reserve remain scarce. As a result, specific metabolites present in FF from patients with POR and their associated metabolic pathways have yet to be clearly defined. Furthermore, their implications for oogenesis, embryo formation, and outcomes related to ART remain largely unclear. This study is the first to employ non-targeted metabolomics technology to identify differential metabolites, elucidate potential metabolic pathways and discover biomarkers in FF associated with POR compared to normal ovarian reserve. This research provides valuable insights for the early diagnosis and clinical intervention of POR.

## 2 Materials and methods

### 2.1 Study population

This study is a case-control pilot study involving 60 women aged between 25 and 38 years who underwent IVF or intracytoplasmic sperm injection at Changzhou Maternal and Child Health Hospital from June 2023 to May 2024. Participants were divided into two groups: the POR group (n=30) and control group (n=30). The diagnosis of POR was based on the Bologna criteria (6): (1) women aged 40 years or older or with other risk factors for POR such as Turner syndrome, a history of ovarian surgery, or prior cancer treatment; (2) poor ovarian response in the previous IVF cycles characterized by obtaining fewer than three oocytes following conventional ovarian stimulation protocols; (3) abnormal ovarian reserve function indicated by antral follicle count (AFC)< 5-7 or anti-Müllerian hormone (AMH) levels< 0.5-1.1 ng/mL. A diagnosis of POR required meeting at least two out of these three criteria.

The control group consisted of patients experiencing infertility due to male infertility or oviduct factors. These individuals were also aged between 25 and 38 years, exhibited normal ovarian reserve function with AMH levels ranging from 2.0 to 6.8 ng/ml, and had an AFC greater than five. Following conventional ovarian stimulation protocols, they obtained more than ten oocytes.

Exclusion criteria for this study included: (1) patients younger than 25 years old or older than 38 years old; (2) any history of ovarian surgery including cystectomy or oophorectomy; (3) conditions such as endometriosis, polycystic ovary syndrome and other disorders that may affect ovarian reserve function; (4) antibiotic treatment within three months prior to participation; (5) any contraindications related to ovulation induction therapy; and (6) systemic abnormalities including chromosomal anomalies, hyperthyroidism, diabetes mellitus, hepatitis B, HIV positivity, systemic lupus erythematosus among others.

The research received approval from the Scientific Research Ethics Committee of Changzhou Maternal and Child Health Care Hospital (No. 2022071), and written informed consent was obtained from each participant.

### 2.2 Sample collection

The individualized ovarian stimulation protocols were tailored to each patient's specific conditions, with concurrent monitoring of follicular development and serum hormone levels. When at least one follicle diameter reaches 18 mm in both ovaries, HCG and/or GnRH-α are used to promote the final maturation of the oocytes after comprehensive evaluation of FSH, E2, and P4 levels.

The FF was aspirated from the first ovarian follicle under transvaginal ultrasound guidance 36 to 38 hours after HCG and/or GnRH-α injection, with confirmation of oocyte presence under the stereomicroscope (Nikon, SMZ1500). After oocyte separation, FF was transferred into a 1.5 mL centrifuge tube and centrifuged at 4°C for 10 minutes at a force of 4×100 g with a centrifugal radius of 8.5 cm. The supernatant was then collected into another 1.5 ml centrifuge tube and collected for frozen preservation in a -80°C refrigerator. The patient's name, age, date and number of retrieved oocytes were recorded. It is important to note that the FF designated for analysis was free from blood contamination.

### 2.3 Metabolomic analysis

The sample preparation involved taking 100 μL of the sample and mixing it with 400 μL of an extraction solution (MeOH: CAN,1:1 v/v), which contained deuterated internal standards. The resulting mixture was vortexed for 30 s, sonicated for 10 min in a water bath maintained at 4°C, and then incubated for 1 h at -40°C to precipitate proteins. Subsequently, the samples were centrifuged at 12,000 rpm (RCF=13,800(×g), R= 8.6 cm) for 15 min at 4°C. The supernatant was carefully transferred to a fresh glass vial for analysis. In addition, pooled quality control (QC) samples were also prepared by combining 10 μL of each extraction mixture to assess the stability of the analytical system.

The extracted samples were randomly sequenced by a Liquid Chromatograph-Mass Spectrometer (LC-MS) system in accordance with established protocols. Initially, chromatographic separations were performed using an ACQUITY UPLC System (Waters, Milford, MA, USA). A Kinetex UPLC C18 column (100mm × 2.1mm, 100A, phenomenex, UK) was used for the reversed-phase separation. The column oven temperature was maintained at 55°C. The flow rate was set to 0.3 mL/min with the mobile phase comprising solvent A (ACN: H2O=6:4, 0.1% formic acid) and solvent B (IPA: ACN=9:1, 0.1% formic acid). Gradient elution conditions were established as follows: 0~0.4 min, 30% B; 0.4~1 min, 30% to 45% B; 1~3 min, 45% to 60% B; 3.5~5 min,60% to 75% B; 5~7 min, 75% to 90% B; 7~8.5 min, 90% to 100% B; 8.5~8.6 min, 100% B; 8.6~8.61 min, 100% to 30% B; 8.61~10 min, 30% B.

A high-resolution tandem mass spectrometer Q-Exactive (Thermo Scientific, Sunnyvale, CA, USA) was utilized for detecting metabolites eluted from the column. The Q-Exactive operated in both positive and negative ion modes. Precursor spectra (70–1050 m/z) were acquired at a resolution of 70,000 to achieve an automatic gain control target of 3e6. The maximum injection time was set to 100 ms. A top three configuration for data acquisition was established in data-dependent acquisition mode. Fragment spectra were collected at a resolution of 17,500 to meet an automatic gain control target of 1e5, with a maximum injection time of 80 ms. To evaluate the stability and consistency of the LC-MS system throughout the acquisition process, a QC sample (a pool of all samples) was collected after every 10 samples. The quality gap between QC samples was used to correct for systematic errors inherent in batch experiments.

### 2.4 Data processing

XCMS software facilitated the identification and alignment of ion peaks across different samples, thereby enabling the retrieval of original abundance information for each metabolic ion present in the samples. MetaX software was employed for data quality control and processing: Low-quality peaks (more than 50% absence in QC samples or more than 80% absence in actual samples) were excluded, and then median normalization was applied to standardize the data. The missing values were addressed through minimum imputation. The raw LC-MS data files were converted into mzXML format and subsequently processed by XCMS along with CAMERA and metaX toolboxes implemented in R software. Each ion was identified by integrating retention time and m/z data. Intensities for each peak were recorded, resulting in the generation of a three dimensional matrix containing arbitrarily assigned peak indices (retention time-m/z pairs), sample names (observations) and ion intensity information (variables).

The KEGG database and Human Metabolome Database (HMDB) was used to annotate the metabolites by matching the exact molecular mass data (m/z) from samples with those those present in these databases. If the mass difference between observed values and database entries was less than 10 ppm, the metabolite would be annotated; further identification and validation of molecular formulas for metabolites were conducted through isotopic distribution measurements. Additionally, we employed an in-house fragment spectrum library of metabolites to corroborate metabolite identification.

### 2.5 Statistical analysis

Principal component analysis (PCA) and orthogonal partial least squares discriminant analysis (OPLS-DA) were performed to illustrate the separation trends between different groups and measure the quality and reliability of the model. Additionally, the variable importance in projection (VIP) for each variable was calculated. The results of PCA and OPLS-DA underwent validation through 200 iterations of 7-fold cross-validation to assess potential overfitting.

For differential metabolite analysis between the two sample groups, univariate analytical methods were used, including fold change (FC) analysis, *t*-tests, and volcano plots. The VIP value served as a metric to evaluate both the strength and explanatory power of each metabolite's expression pattern concerning classification discrimination across sample groups. Metabolites exhibiting FC≥1.2 or FC ≤ 1/1.2, *P*<0.05 and VIP≥1 were selected as significantly different metabolites.

The classification and annotation information from HMDB and KEGG databases facilitated the functional annotation of identified metabolites while corresponding graphs were generated accordingly. Hypergeometric-based enrichment analyses with KEGG Pathways were conducted; *P* values for these pathways primarily derived from hypergeometric tests. Spearman correlation analysis was used to investigate the relationships between differential metabolites and clinical indices.

Prediction probabilities were computed using random forest and logistic regression models. The receiver operating characteristic (ROC) curves were generated to determine 95% confidence intervals along with area under the curve (AUC). Data analysis and graphical representations were executed using OmicStudio (https://www.omicstudio.cn/tool) based on the R implementations of relevant algorithms.

To justify the sample size, we employed PASS 15.0 software based on the results of the metabolomic profiling. The required sample sizes for differential metabolites, with a significance level set at 0.05 and a power of 0.90, are detailed in **Supplementary Table S1**. Regarding participants' clinical characteristics: R4.4.2 was used to test data normality and homogeneity. The data conforming to normal or near normal distributions with homogeneity of variance were analyzed by independent sample *t* test and expressed as mean ± standard deviation. In cases where these assumptions were not met, the Mann-Whitney *U* test was employed and statistical descriptions utilized median and quartile values. Frequencies were compared using the Chi-square test. *P ≤* 0.05 was considered statistically significant.

## 3 Results

### 3.1 Clinical characteristics of patients with POR

A total of 60 participants were included in this metabolomic study, comprising 30 individuals in the control group and 30 in the POR group. The clinical characteristics of these participants are shown in **Table 1**. The mean age of the participants was 32.77 ± 2.79 years, with ages ranging from 25 to 37 years. No significant differences were observed between the POR group and control group regarding age, duration of infertility, primary infertility rate, baseline luteinizing hormone (bLH) levels, baseline estradiol (bE2) levels, or testosterone (T) levels (*P*>0.05).

**Table 1:** Clinical characteristics of controls and POR group.

Variables	POR group (n=30)	Control group (n=30)	*P* value
Age (year)	32.77 ± 2.88	32.77 ± 2.74	1.000
bFSH (mIU/mL)	8.34 (7.54,15.34)	6.09 (5.51,6.95)	<0.001^***^
bLH (mIU/mL)	3.96 (2.98,6.27)	4.83 (3.89,6.28)	0.203
bE2 (pg/mL)	32.42 (22.71,52.13)	41.30 (33.88,47.10)	0.176
T (ng/mL)	22.36 (16.48,28.60)	25.44 (20.28,38.18)	0.739
AFC (n)	3.07 ± 1.82	11.53 ± 4.73	<0.001^***^
AMH (ng/mL)	0.29 ± 0.23	3.81 ± 1.34	<0.001^***^
Duration of infertility (year)	4.00 (2.00,5.00)	2.00 (1.50,4.00)	0.051
Primary infertility ratio (%)	66.67 (20/30)	46.67 (14/30)	0.193
Oocytes retrieved (n)	1.40 ± 0.50	18.03 ± 4.73	<0.001^***^
MII oocytes (n)	1.30 ± 0.60	15.50 ± 4.18	<0.001^***^
2PN Fertilizations (n)	1.00 ± 0.74	11.63 ± 4.48	<0.001^***^
Day3 High-quality embryos (n)	0.73 ± 0.69	8.90 ± 5.00	<0.001^***^

bFSH, basic follicle stimulating hormone; bLH, basic luteinizing hormone; bE2, basic estrogen; T, Testosterone; AFC, antral follicle count; AMH, anti-Mullerian hormone. *** *P*<0.001.

However, the level of basic follicle stimulating hormone (bFSH) in POR group was significantly higher than that in control group (*P*<0.001). Additionally, parameters such as bAFC, AMH levels, number of oocytes retrieved, metaphase II (MII) oocytes count, fertilization, and Day3 high-quality embryos were all significantly lower in patients with POR compared to those in control group (*P*<0.001), indicating markedly reduced ovarian function among POR patients.

### 3.2 Multivariate analysis of metabolites

In this research, we investigated metabolites present in FF under both positive and negative ion modes. A total of 10,763 variable features were detected, leading to the identification of 1470 metabolites; specifically,780 metabolites were identified in the positive ion mode while 690 metabolites were identified in the negative ion mode.

**Figure 1:**
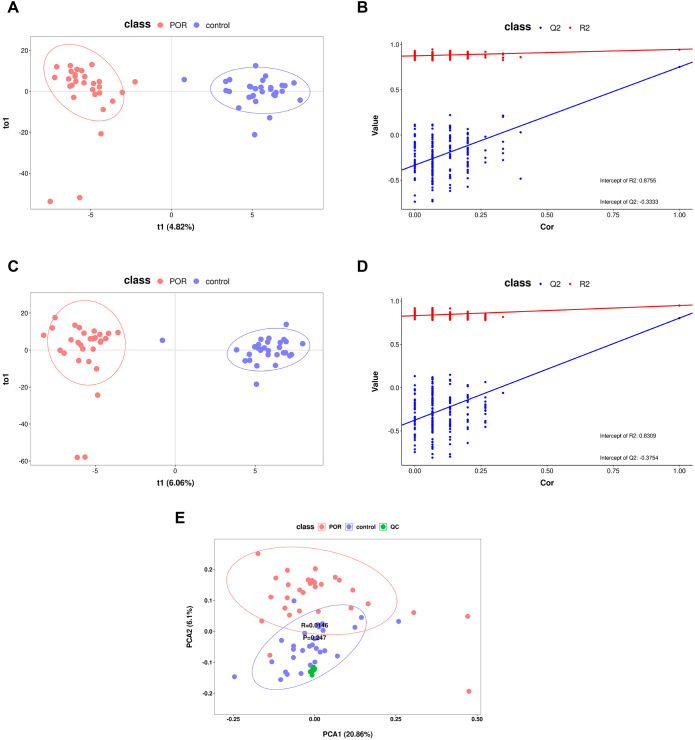
OPLS-DA and PCA score graphs with corresponding permutation test plots. **(A)** OPLS-DA score plots for positive ionization mode derived from data obtained from POR and control FF samples; **(B)** the permutation test results of the OPLS-DA model for positive ionization mode; **(C)** OPLS-DA score plots for negative ionization mode based on data from POR and control FF samples; **(D)** the permutation test results of the OPLS-DA model for negative ionization mode; **(E)** PCA score graph encompassing all samples along with QC samples.

Utilizing metabolite data obtained from both ion modes, OPLS-DA and PCA models were established to compare overall metabolic profile differences among patients with POR, control subjects, and QC samples. As illustrated in **Figures 1A, C**, both positive and negative ion modes demonstrated that the OPLS-DA model effectively separated the POR group from the control group; they exhibited distinct clustering patterns—wherein the POR group was uniformly clustered on the left side while the control group was consistently clustered on the right side. **Figure 1E** further indicates that QC samples grouped closely together and remained well-separated from both POR group and control groups, thereby confirming stability and repeatability within our detection system. However, it is noteworthy that complete separation between POR and control groups was not achieved; this suggests individual variability among samples.

Simultaneously, results from OPLS-DA underwent validation through a rigorous process involving 200 iterations of seven-fold cross-validation alongside a permutation test model designed to assess potential overfitting issues within our model framework. As depicted in **Figures 1B, D**—the abscissa ranges from [0 to1], with R^2^ regression lines positioned above Q^2^—and an intercept between Q^2^ regression line along with Y-axis being less than zero—these findings indicate no evidence of overfitting within our model while reflecting its robust simulation efficacy as well as predictive capability for OPLS-DA analysis.

### 3.3 Overall metabolic profile and differential metabolites in POR

**Figure 2:**
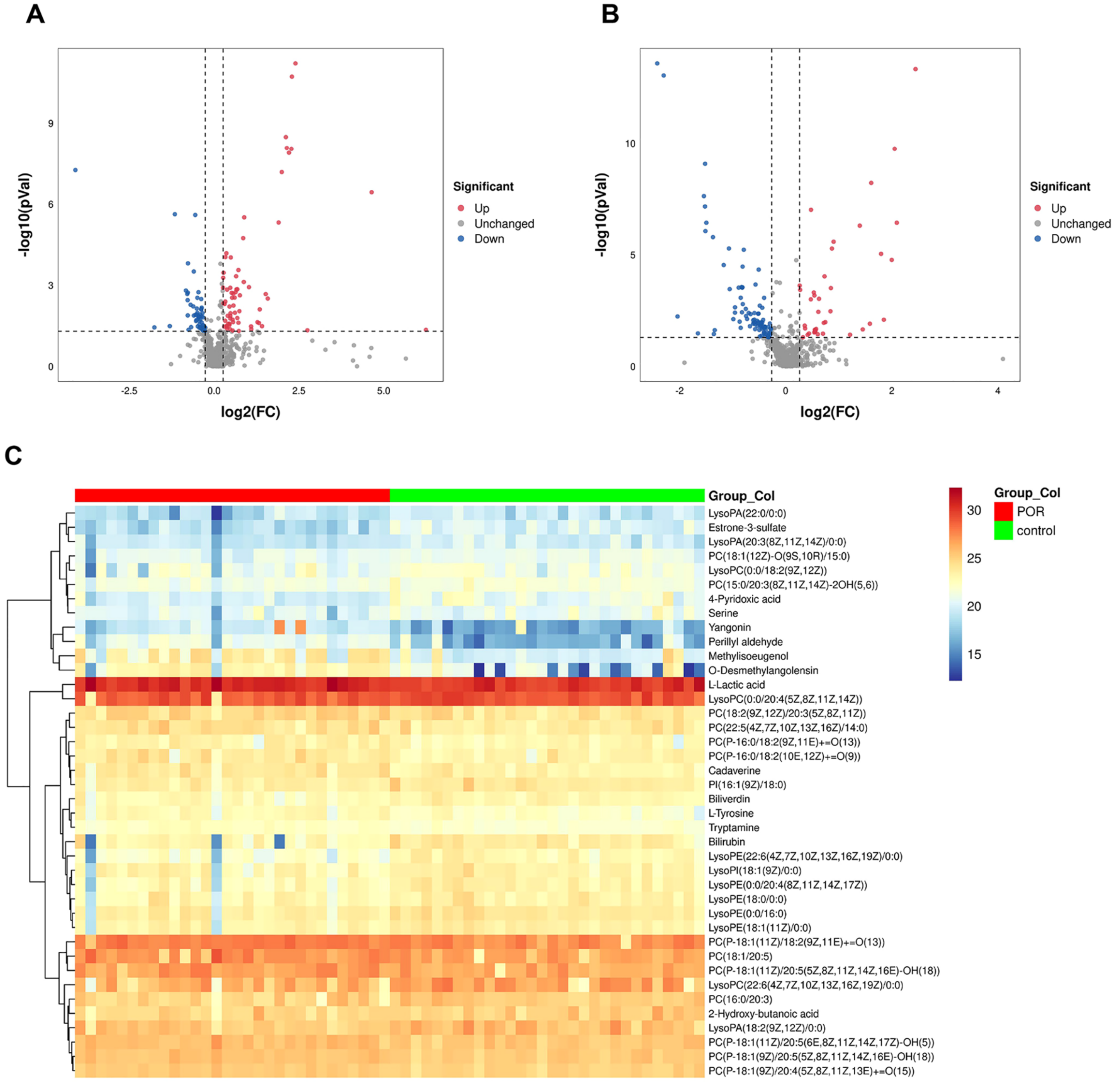
Volcano plot illustrating differential metabolites across various ion modes. Blue dots signify up-regulated features, while red dots denote down-regulated features. The down-regulated metabolites in POR samples are predominantly clustered on the left, whereas up-regulated metabolites are primarily found on the right side. **(A)** positive ionization mode; **(B)** negative ionization mode; **(C)** Heatmap representing 40 differential metabolites.

The FC of metabolites between groups was analyzed. The abundance values of the metabolites were log2 transformed to ensure that the data conformed to a normal distribution, followed by performing a *t*-test. The VIP value for each metabolite was derived from multivariate statistical OPLS-DA analysis, allowing for the identification of differentially expressed metabolites. To be considered significant, differential metabolites had to meet the criteria: FC≥1.2 or FC ≤ 1/1.2, *P*<0.05, and VIP≥1 simultaneously. A total of 221 metabolites were identified as differing between the two groups. Volcano plots illustrating these differential metabolites in both positive and negative ion modes are presented in **Figures 2A, B**; down-regulated metabolites in POR samples are clustered on the left side while up-regulated metabolites are clustered on the right side.

Among these, 40 metabolites were found to be present in both HMBD database and KEGG database. A heat map depicting these 40 differential metabolites is shown in **Figure 2C**, visually representing changes in metabolite levels among POR patients.

According to classifications based on the HMBD database, these identified metabolites included glycerophospholipids, benzene and their substitutes, carboxylic acids and their derivatives, hydroxy acids and their derivatives, indoles and their derivatives, organonitrogen compounds, tetrapyrroles along with their derivatives, steroids as well as steroid derivatives.

In comparison with the control group, there were significantly elevated levels of 18 specific metabolites while levels of another 22 distinct metabolites were notably reduced within FF from patients with POR. Among those that exhibited increased expression were l-lactic acid, methylisoeugenol, l-tyrosine, o-desmethylangolensin, yangonin, perillyl aldehyde, cadaverine, tryptamine, and most phosphatidylcholine (PC). Conversely, the down-regulated substances included estrone-3-sulfate, 4-pyridoxic acid, serine, 2-hydroxy-butanoic acid, lysolecithin, phosphatidylinositol (PI), bilirubin and biliverdin.

### 3.4 Correlation analysis between differential metabolites and clinical indices

Spearman correlation analysis was employed to investigate the relationship between differential metabolites in FF and clinical indices. A correlation coefficient (rho) greater than 0.5, accompanied by a *P*-value of less than 0.05, indicated that a significant correlation between the two indices.

**Figure 3:**
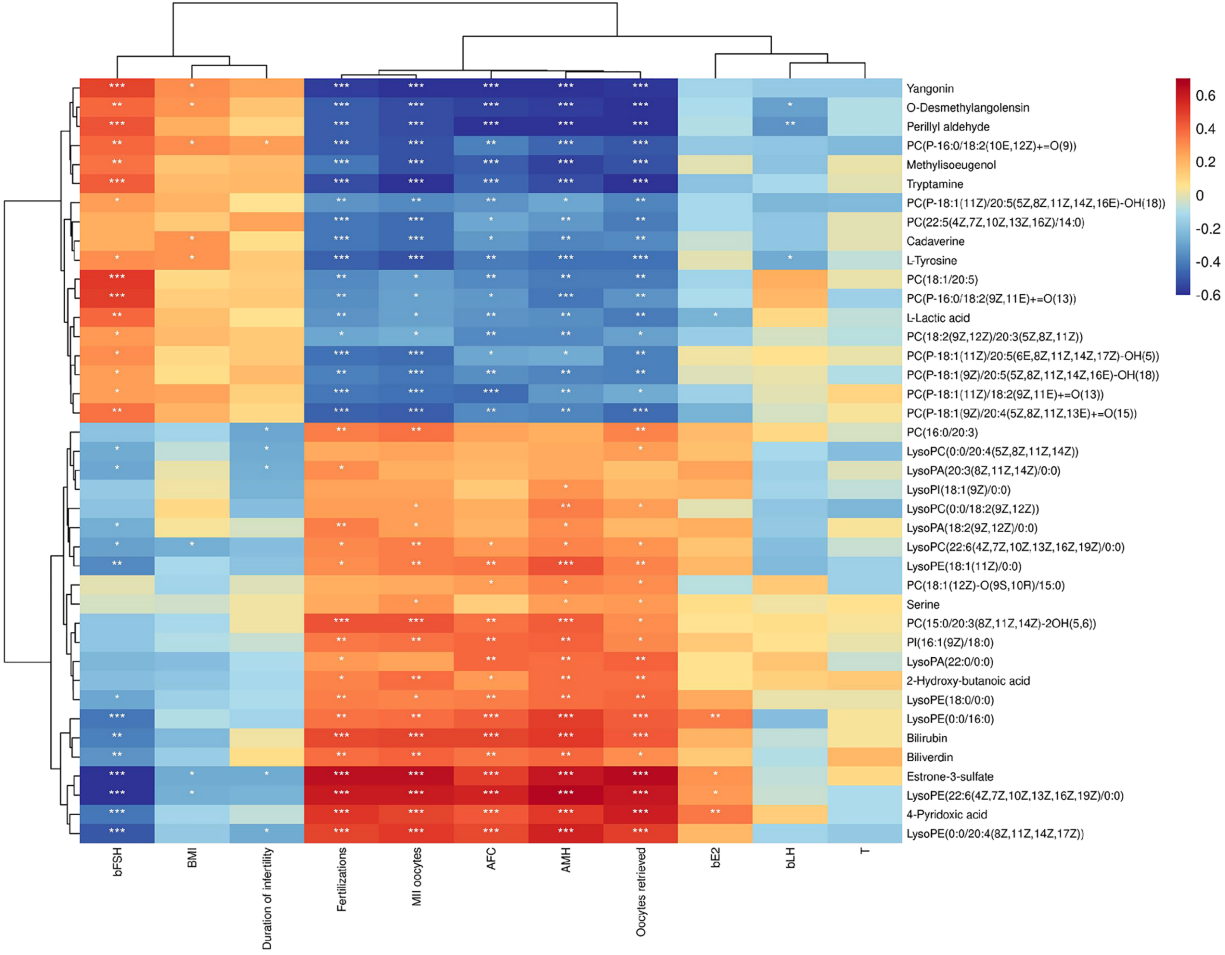
Correlation Clustering Heatmap. The names of metabolites are displayed on the right side of the figure, while clinical indices are listed at the bottom. Each grid within this figure represents the correlation between two attributes—metabolites and hormones—with varying colors indicating different the magnitudes of correlation coefficients between these attributes. *P < 0.05, **P < 0.01, ***P < 0.001.

As illustrated in **Figure 3**, we observed that methylisoeugenol, o-desmethylangolensin, yangonin, tryptamine and perillyl aldehyde exhibited significant negative correlations with AMH, the number of oocytes retrieved, and MII oocytes. Conversely, estrone-3-sulfate, 4-pyridoxic acid and lysophosphatidylethanolamine (22:6 (4Z,7Z,10Z,13Z,16Z,19Z) /0:0) demonstrated positive correlations with the number of oocytes retrieved as well as AMH levels and 2PN fertilization. However, most metabolites did not show any significant correlations with body mass index, bLH, T, bE2, or duration of infertility; all had rho values below 0.4.

### 3.5 Metabolic pathway analysis

**Figure 4:**
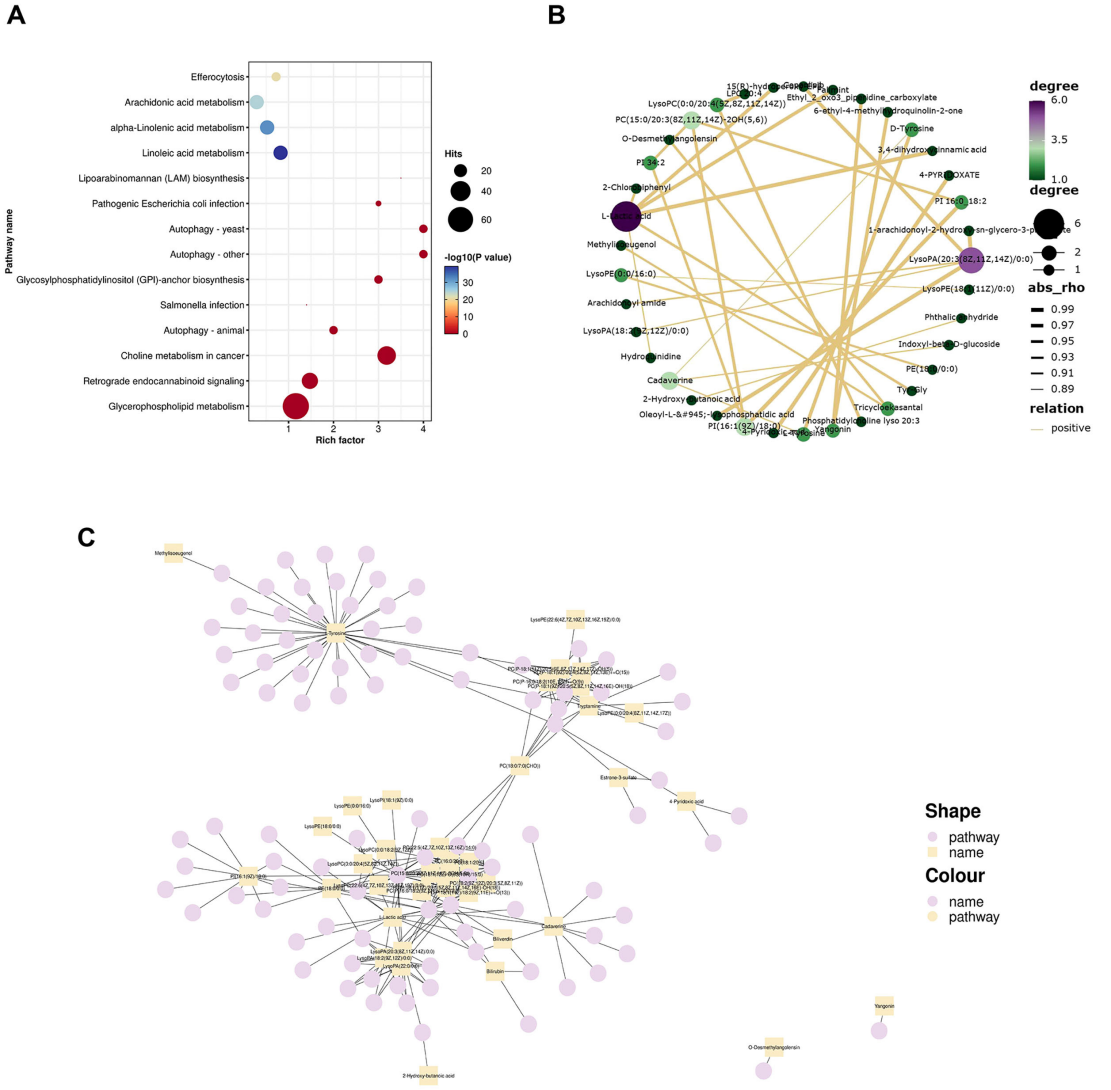
Metabolic pathway analysis. **(A)** Bubble plot depicting KEGG enrichment analysis results. The top twenty pathways based on *P* values were utilized to create this bubble diagram display. The Rich Factor indicates the ratio of differential metabolites present in a given pathway to the total number of metaboliteswithin that pathway—the larger this value, the greater degree of enrichment associated with that pathway is indicated by dot size, which corresponds to both quantity and significance: larger dots represent more differential metabolites in each pathway, while color variations reflect *P* values related to enrichment analysis significance; **(B)** Correlation network of the top 30 metabolites based on *P* values. Each point represents a distinct substance. The greater the number of lines connecting a point, the more associated objects there are, indicating that more metabolites may be influenced; **(C)** Network plot illustrating 40 differential metabolites and their corresponding metabolic pathways. Squares denote differential metabolites while circles represent metabolic pathways. An increased number of connections to a point signifies a higher degree of association with other entities, suggesting that specific pathways and certain metabolites are central to the regulatory mechanism.

KEGG pathway analysis revealed that the differential metabolites were primarily involved in glycerophospholipid metabolism, choline metabolism in cancer, autophagy, glycosylphosphatidylinositol-anchor biosynthesis, lipoarabinomannan biosynthesis, pathogenic Escherichia coli infection, Salmonella infection, retrograde endocannabinoid signaling, etc (*P*<0.05). These metabolic pathways are crucial for maintaining normal cellular and tissue function as depicted in **Figure 4A**.

The top thirty significant differential metabolites based on *P* value were selected for further analysis to elucidate their intercorrelations derived from the relationships among these metabolites. Relationship pairs exhibiting |rho|>0.7 were filtered to construct a correlation network shown in **Figure 4B**. Notably, L-lactic acid along with lysophosphatidic acid (20:3(8Z,11Z,14Z)/0:0) and PC [15:0/20:3(8Z,11Z,14Z)-2OH (5, 6)] displayed associations with numerous other metabolites, indicating their close interrelationship within this metabolic context. This finding further underscores alterations in metabolic profile present within FF samples from patients diagnosed with POR.

According to the KEGG information regarding differential metabolites, a network plot illustrating the relationship between 40 differential metabolites and their associated pathways was generated, as depicted in **Figure 4C**. Notably, tryptamine, PC, PI, lysophosphatidic acid, lysophosphatidylcholine (LPC), l-tyrosine, cadaverine and l-lactic acid were involved in the greatest number of pathways, indicating that their central role in metabolic processes.

### 3.6 Potential biomarker analysis

Among the 40 differential metabolites identified, those with a VIP value greater than 1.5 were selected as potential biomarkers. It is important to note that while the VIP value within the OPLS-DA model represents its separation capability, it alone does not suffice to determine whether these selected metabolites are appropriate for diagnosing POR. Therefore, additional statistical criteria— including FC, *P-*value, and q-value—must be comprehensively evaluated; this is summarized in **Table 2**.

**Table 2:** VIP values of metabolites to be used as potential biomarkers.

Metabolite	VIP	Fold change	*P* value	Q value
Perillyl aldehyde	4.43	4.15	<0.0001	<0.0001
Estrone-3-sulfate	2.76	0.35	<0.0001	<0.0001
Yangonin	3.76	24.98	<0.0001	<0.0001
Methylisoeugenol	3.37	4.27	<0.0001	<0.0001
4-Pyridoxic acid	2.39	0.35	<0.0001	<0.0001
O-Desmethylangolensin	4.16	3.74	<0.0001	0.0002
LysoPE(22:6(4Z,7Z,10Z,13Z,16Z,19Z)/0:0)	2.40	0.45	<0.0001	0.0010
L-Tyrosine	1.78	1.64	0.0003	0.0056
LysoPE(0:0/20:4(8Z,11Z,14Z,17Z))	1.96	0.57	0.0003	0.0059
Cadaverine	1.52	1.55	0.0080	0.0230

VIP, variable importance on projection.

**Figure 5:**
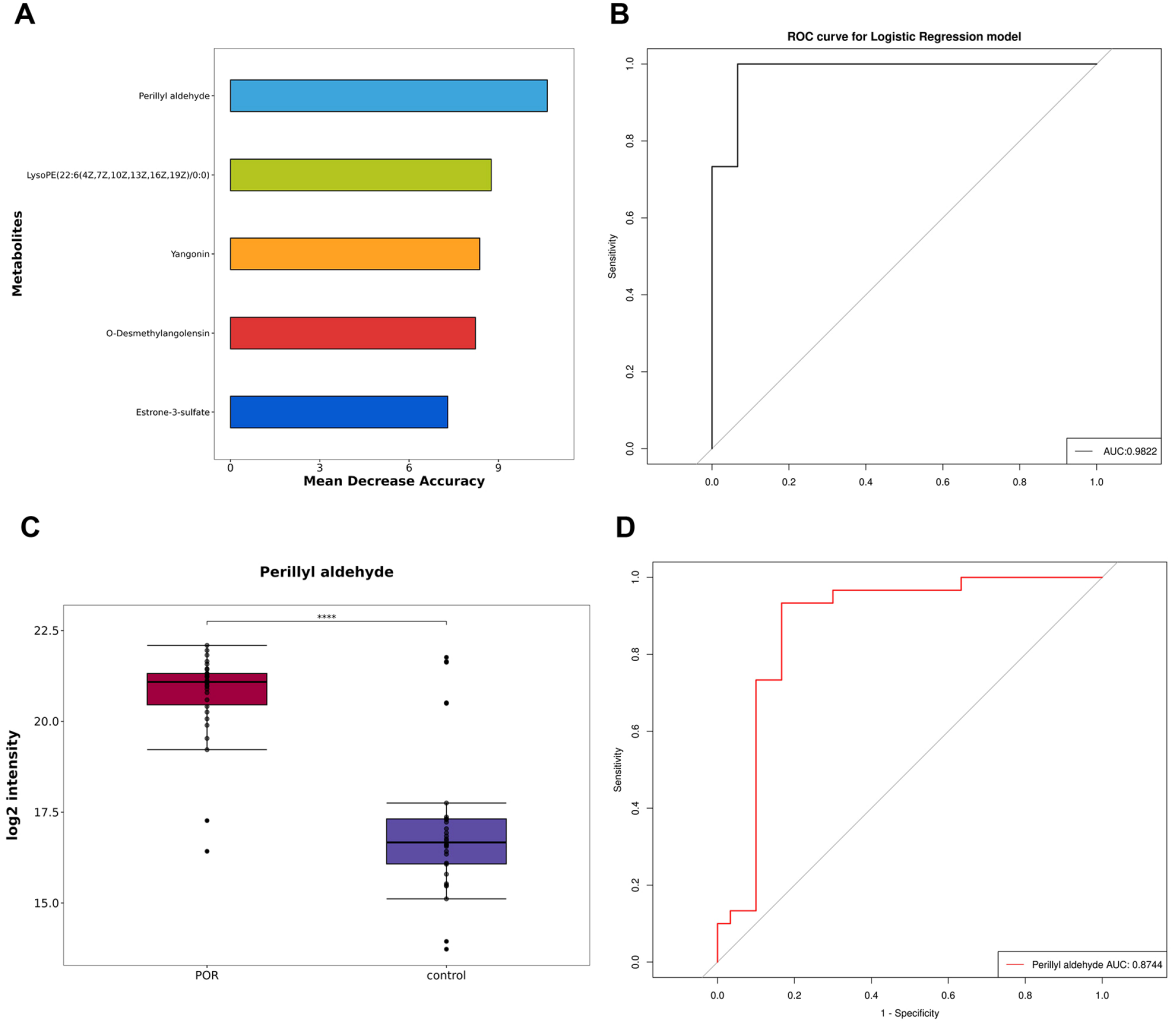
Establishment and test of diagnostic model. **(A)** The top five metabolites selected using the random forest algorithm; **(B)** ROC curve and AUC values for the combined diagnostic model derived from these top five metabolites; **(C)** Box plot depicting perillyl aldehyde; **(D)** ROC curve and AUC values for perillyl aldehyde ****P<0.0001.

Furthermore, random forest analysis was conducted where mean decrease accuracy and mean decrease Gini were employed to assess metabolite importance. As illustrated in **Figure 5A**, the top five most important metabolites identified through random forest analysis include perillyl aldehyde, LPC(22:6(4Z,7Z,10Z,13Z,16Z,19Z)/0:0), yangonin, o-desmethylangolensin, and estrone-3-sulfate. The ROC curve was used to evaluate the diagnostic performance of our model, as shown in **Figure 5B**. The AUC was calculated at 0.9822, indicating excellent predictive efficacy and suggesting these five metabolites could serve as reliable indicators for ovarian function assessment.

Additionally, **Table 2** and **Figures 5A, B** demonstrate that perillyl aldehyde possesses substantial diagnostic value and may be considered a potential marker for POR diagnosis. The box plot and ROC curve pertaining to perillyl aldehyde are displayed in **Figures 5C, D** respectively. The AUC for perillyl aldehyde reached an impressive score of 0.8744 with a corresponding confidence interval of [0.772, 0.9769].

## 4 Discussion

FF constitutes a complex biochemical microenvironment that orchestrates oocyte growth and follicular maturation. In contrast to serum or urine, FF exhibits a rich composition comprising steroid hormones, amino acids, reactive oxygen species, and antioxidant enzymes, all of which play critical roles in regulating oocyte maturation, fertilization competence, and early embryonic development (12). Consequently, metabolomic profiling of FF provides direct insights into the molecular determinants governing oocyte developmental potential. Previous metabolomic analyses conducted on serum samples from POR patients by Song et al. implicated steroid hormone biosynthesis and tyrosine metabolism in POR pathogenesis, aligning with our results (13). In this study, we employed untargeted metabolomics to analyze FF obtained from 60 subjects, identifying 18 significantly upregulated and 22 downregulated metabolites in POR patients compared to controls. These differential metabolites were predominantly enriched in choline metabolism, glycerophospholipid metabolism, and autophagy pathways. Notably, ROC analysis identified perillyl aldehyde as a potential biomarker for POR. Our results expand upon existing evidence by providing deeper mechanistic insights into POR.

PC is the most abundant phospholipid found in eukaryotic cells and tissues synthesized through the choline pathway (14). PC serves as a precursor for sphingomyelin and prostaglandins, which play critical roles in mediating inflammatory responses (15). Furthermore, choline can be converted into platelet-activating factor synthetase, thereby facilitating the production of platelet-activating factor—a key mediator of inflammation and angiogenesis (16). Consequently, PC is closely linked to inflammatory process. Increasing evidence suggests that chronic low-grade inflammation can alter the follicle environment and lead to elevated levels of intracellular reactive oxygen species that impair oocyte function (17) and negatively affect ART outcomes.

In our study, we observed an increase in PC levels within the FF of POR patients, indicating heightened levels of inflammatory mediators present in their FF. This suggests that the local microenvironment within the FF of individuals with POR may exist in a state characterized by chronic inflammation.

Lysophospholipids are small molecular glycerophospholipids known for their strong surface-active properties; they also serve as important extracellular signalling molecule. Studies have demonstrated that LPC promotes oocyte maturation and granulosa cell proliferation by activating nitric oxide pathways and extracellular signal-regulated kinase signalling cascades (18). Moreover, exogenous administration of LPC has been shown to significantly mitigate the inhibitory effects exerted by zearalenone on oocyte development and maturation (19). In addition, the incorporation of lysophosphatidicacid (LPA) into the *in vitro* culture system has been shown to significantly enhance the maturation rate of bovine oocytes (20). Another study demonstrated that LPA could improve oocyte quality during ART by activating ERK1/2 pathway in both granulosa cells and oocytes (21). Furthermore, low levels of lysophosphatidylinositol (LPI) has been associated with a reduced number of retrieved and mature oocytes. LPI has been found to inhibit hemin-induced oxidative stress in granulosa cells and can partially reverse hemin-induced cell proliferation inhibition, senescence and apoptosis (22).

In this study, we observed that the levels of LPA, LPC, LPI, and lysophosphatidylethanolamine were significantly decreased in the FF of patients with POR. Additionally, there was a positive correlation between the number of fertilized oocytes and high-quality embryos with metabolites derived from LPC. This suggests that lysophospholipids present in FF play a crucial role in determining oocyte quality and embryo development; thus, their reduction may contribute to dysplasia in granulosa cells as well as disorders related to oocyte maturation.

Lactate, an end product of glycolysis, serves as an important metabolic fuel source and gluconeogenic precursor. Research indicates that elevated lactate levels are negatively correlated with overall oocyte and embryo quality as well as the fertilization rates (23). High lactate concentrations within FF serve as significant indicators of follicular dysgenesis. Karaer et al. (24) reported markedly increased levels of lactate and pyruvate in the FF from patients suffering from endometriosis; these elevations were linked to inhibited follicular development. Marianna et al. (25) discovered that increased lactate levels in FF of patients with endometriosis may negatively impact follicular development. Furthermore, the increased production of lactate in FF could lead to a decrease in its pH. Under normal physiological conditions, FF is characterized by an alkaline environment; thus, a reduction in pH may result in an acid-base imbalance within the FF, ultimately diminishing oocyte fertilization rates (26).

In the present study, FF from patients with POR exhibited decreased glucose levels alongside increased lactate concentrations, both of which are associated with glycolysis and can serve as energy sources for oocyte maturation. The alteration of these three metabolites underscores the activation of the glycolytic pathway. The enhanced glycolytic activity may be attributed to heightened granule cell activity; notably, lactate is one of the primary metabolic products produced by these cells (27). The observed increase in lactate levels primarily results from augmented glycolysis within granule cells (24), which occurs as a compensatory mechanism to meet energy demands under local hypoxic conditions (28).

Biliverdin (BV), a product derived from heme catabolism, can be swiftly converted into bilirubin by BV reductase. Both BV and bilirubin—when maintained within normal physiological ranges—exhibit various beneficial effects on health. Studies have indicated that BV can diminish oxidative stress markers within brain tissue and mitigate DNA damage following ischemia-reperfusion injury in rat models; this action contributes to reduced cerebellar infarction volume and serves protective roles against cellular damage (29). In humans, glutathione plays a crucial role in safeguarding water-soluble proteins from reactive oxygen species (ROS) via participation in the glutathione cycle; similarly, BV functions as part of the BV-bilirubin cycle to protect lipids from ROS exposure (30). Additionally, BV possesses certain anti-inflammatory properties: under synergistic influence with bilirubin reductase, it can inhibit inflammatory responses through modulation of the endothelial nitric oxide synthase/nitric oxide/toll-like receptor-4 pathway (31). In addition, BV can regulate cell apoptosis, and its mechanism may be associated with the scavenging of oxygen free radicals, which affects mitochondrial function and inhibits the expression of apoptosis-related proteins (32).

BV can be catalyzed by BV reductase to produce bilirubin, an endogenous antioxidant with cytoprotective properties. Numerous studies have demonstrated that the antioxidant and anti-inflammatory activities resulting from mildly elevated bilirubin levels can play a protective role in the human body (33). Bilirubin acts as an endogenous antioxidant that removes excessive ROS and prevents DNA, proteins, and membranes caused by ROS accumulation (34).

The present study observed decreased levels of BV and bilirubin in FF of patients with POR were decreased, which may lead to an imbalance between local oxidation and antioxidation within FF, consequently inducing oxidative stress (OS). OS has been linked to a reduction in ovarian reserve (35), further contributing to POR.

Autophagy is a fundamental molecular pathway essential for maintaining cellular and organismal homeostasis. The formation of autophagosomes along with the degradation of organelles and cytoplasmic proteins via lysosomal pathways serves to uphold cellular stability across tissues and organisms (36). Under physiological conditions, autophagy plays a protective role within the body. Both ROS and OS can activate autophagy; this process protects cells through negative feedback mechanisms while selectively eliminating sources of ROS (37), thereby preserving granulosa cell stability. Typically, granulosa cells sustain ovarian function and regulate follicular development through autophagic homeostasis. Recent studies have indicated that granulosa cell autophagy maintains citrate levels during oocyte maturationby selectively targeting ATP citrate lyase, thus regulating normal follicle development while ensuring internal environmental homeostasis (38).

Mitochondria are the most abundant organelles in oocytes and play a crucial role in mediating the synchronous development of both the nucleus and cytoplasm within these cells. Consequently, the normal structure and stable function of mitochondria are regarded as significant indicators of follicular maturation (39). Granulosa cells serve as essential supporting cells for oocytes; their growth, proliferation, division, and steroidogenesis necessitate an ample supply of functional and stable mitochondria to meet energy demands (40). Therefore, structurally sound, functionally robust, and plentiful mitochondria are vital for the proper functioning of the ovary. Under normal physiological conditions, moderate mitophagy occurs in ovarian cells to prevent the accumulation of damaged mitochondrial DNA (mtDNA) and to inhibit the transmission of abnormally shaped mitochondria to offspring. A reduction or excessive activation of mitophagy can result in mitochondrial dysfunction that adversely affects oocyte development and granulosa cell viability, ultimately contributing to ovarian aging (41). Moreover, mitochondria represent the primary site for oxidative reactions within the body; this process is accompanied by ROS production. However, an overabundance of ROS can lead to mitochondrial damage (42), trigger apoptotic factors release, and induce cell death. Selective autophagy not only aids in ensuring survival for ovarian granulosa cells but also helps maintain ROS levels at relatively low concentrations by eliminating damaged mitochondria from compromised granulosa cells (43).

In this study, we observed that various autophagy pathways were down-regulated in FF of patients with POR, leading to a diminished capacity for cell degradation and recycling. Consequently, the levels of ROS in FF increased. A significant accumulation of damaged organelles and degradation products could not be effectively cleared by autophagy, resulting in an impaired FF environment that exacerbates OS and mitochondrial dysfunction, ultimately affect the development and maturation of follicles.

Furthermore, our findings indicate that perillyl aldehyde may serve as a potential biomarker for POR. Perillyl aldehyde is a volatile natural monoterpenoid compound derived from perilla. It has been reported to possess substantial antioxidant properties, enhancing tissue cells defend against OS. Some researchers have demonstrated that perillyl aldehyde can inhibit the aryl hydrocarbon receptor signaling pathway while activating the nuclear factor E2-related factor 2 antioxidant pathway in human keratinocytes; this action subsequently inhibits ROS production and mitigates OS induced by environmental pollutants (44). Additionally, Candida albicans infection within vaginal tissues of mice can lead to excessive ROS accumulation and subsequent cell apoptosis. Perillyl aldehyde has been shown to suppress NADPH2 oxidase expression in vaginal tissues while increasing antioxidant enzyme activity to reduce ROS levels, thereby alleviating tissue damage (45).

This study revealed that the level of perillyl aldehyde in the FF of patients with POR was significantly up-regulated. Additionally, we observed a notable negative correlation between the concentration of perillyl aldehyde in the FF and both AMH levels as well as the number of oocytes retrieved, including MII oocytes. These findings suggest that OS within the microenvironment of FF in POR patients may trigger an activation of the antioxidant system *in vivo*, leading to an increase in perillyl aldehyde. Consequently, in clinical applications, perillyl aldehyde may serve as a biomarker of OS within the FF microenvironment associated with POR. The level of perillyl aldehyde could potentially reflect the severity of POR. Moreover, following appropriate pharmacological processing, it may be utilized therapeutically due to its antioxidant properties to improve ART outcomes. However, given the limitations imposed by sample size, further exploration is needed regarding the effects of perillyl aldehyde on oocyte development and its clinical predictive value.

We used untargeted metabolomics to characterize metabolic differences in FF between individuals with POR and those with normal ovarian reserve for the first time. Our metabolic profiling reveals novel mechanistic insights into the pathogenesis of POR.This study does have several limitations. The small sample size may lead to insufficient statistical significance that affects the generalizability of our findings. As a single-center cross-sectional study, we cannot establish causality from our observations; thus, these results may not be applicable to other centers or broader populations. Future research should employ larger sample sizes and multicenter study designs to enhance the generalizability of findings. Additionally, FF was collected during ovarian stimulation which could alter its metabolic profile and therefore might not represent its natural state. While we controlled for age and iatrogenic factors, the lack of subgroup analyses based on etiology or markers of ovarian reserve limits our understanding of subgroup-specific metabolic variations.

This study identified several metabolic pathways associated with the progression of POR, including glycerophospholipid metabolism, choline metabolism, and autophagy. However, direct experimental validation remains absent. Notably, Wu et al. (46) conducted pseudotargeted metabolomic analysis of FF and revealed dysfunction in glycerophospholipid metabolism mediated by GPD1L in patients with decreased ovarian reserve. Furthermore, through a comparative metabolomic analysis of FF from infertile patients with polycystic ovary syndrome or diminished ovarian reserve, Shen et al. (47) identified significant enrichment of choline metabolism pathways in individuals with diminished ovarian reserve. Yao et al. (48) demonstrated that Nur77 overexpression induces mitochondrial autophagy, effectively counteracting oxidative stress while preserving ovarian reserve in murine models of reproductive aging. In our study, we observed that both glycerophospholipid metabolism and choline metabolism are implicated in the POR group, alongside observed downregulation of various autophagy pathways within the FF of these patients. These findings align with those previously reported and provide substantial support for our initial hypothesis. Looking ahead, *in vivo* and *in vitro* experimental validations should be conducted to elucidate the relationships between POR and these metabolic pathways. In addition, due to the current limitations in technology, we are unable to conduct a targeted metabolomic analysis or validate the dataset pertaining to perillyl aldehyde at this time. As analytical technologies mature, targeted metabolomic profiling of perillyl aldehyde could be implemented to substantiate these preliminary findings. Moreover, a robust framework for elucidating the mechanistic profile of perillyl aldehyde will be established, which is to employ integrated cellular and animal models to systematically evaluate its regulatory mechanisms on granulosa cells while assessing potential clinical applications.

## Data Availability

The raw data supporting the conclusions of this article will be made available by the authors, without undue reservation.
