# Supplementary material for: Non-targeted metabolomic analysis of follicular fluid in infertile individuals with poor ovarian response
Source: Front Endocrinol (Lausanne). 2025 May 12;16:1547550. doi: 10.3389/fendo.2025.1547550 (PMC12104088; doi:10.3389/fendo.2025.1547550)
Supplement: Supplementary file 1 [file Table1.docx]

**Table 1. Sample size for 40 differential metabolites with the level of significance set at 0.05 and the power at 0.90.**

| **Metabolite** | **Sample size/per group** |
| --- | --- |
| LysoPE(22:6(4Z,7Z,10Z,13Z,16Z,19Z)/0:0) | 10 |
| O-Desmethylangolensin | 10 |
| Perillyl aldehyde | 10 |
| Tryptamine | 11 |
| Estrone-3-sulfate | 13 |
| L-Tyrosine | 14 |
| LysoPE(0:0/20:4(8Z,11Z,14Z,17Z)) | 16 |
| Methylisoeugenol | 17 |
| PC(P-18:1(9Z)/20:4(5Z,8Z,11Z,13E)+=O(15)) | 20 |
| Cadaverine | 21 |
| PC(P-18:1(9Z)/20:5(5Z,8Z,11Z,14Z,16E)-OH(18)) | 21 |
| Bilirubin | 22 |
| PC(P-16:0/18:2(10E,12Z)+=O(9)) | 23 |
| 4-Pyridoxic acid | 23 |
| LysoPE(0:0/16:0) | 25 |
| PC(18:2(9Z,12Z)/20:3(5Z,8Z,11Z)) | 27 |
| PC(P-16:0/18:2(9Z,11E)+=O(13)) | 31 |
| PC(P-18:1(11Z)/18:2(9Z,11E)+=O(13)) | 33 |
| PC(P-18:1(11Z)/20:5(5Z,8Z,11Z,14Z,16E)-OH(18)) | 34 |
| PC(P-18:1(11Z)/20:5(6E,8Z,11Z,14Z,17Z)-OH(5)) | 34 |
| LysoPC(22:6(4Z,7Z,10Z,13Z,16Z,19Z)/0:0) | 36 |
| PC(18:1/20:5) | 38 |
| LysoPA(20:3(8Z,11Z,14Z)/0:0) | 39 |
| PC(22:5(4Z,7Z,10Z,13Z,16Z)/14:0) | 40 |
| PC(15:0/20:3(8Z,11Z,14Z)-2OH(5,6)) | 42 |
| Biliverdin | 42 |
| LysoPE(18:1(11Z)/0:0) | 46 |
| L-Lactic acid | 49 |
| PC(18:1(12Z)-O(9S,10R)/15:0) | 51 |
| LysoPA(18:2(9Z,12Z)/0:0) | 52 |
| LysoPA(22:0/0:0) | 54 |
| LysoPE(18:0/0:0) | 55 |
| PI(16:1(9Z)/18:0) | 58 |
| 2-Hydroxy-butanoic acid | 61 |
| LysoPC(0:0/20:4(5Z,8Z,11Z,14Z)) | 68 |
| PC(16:0/20:3) | 70 |
| LysoPI(18:1(9Z)/0:0) | 80 |
| Serine | 84 |
| LysoPC(0:0/18:2(9Z,12Z)) | 139 |
| Yangonin | 148 |
